# Gut Microbiota Dysbiosis and Dietary Interventions in Non-Hodgkin B-Cell Lymphomas: Implications for Treatment Response

**DOI:** 10.3390/biomedicines13092141

**Published:** 2025-09-02

**Authors:** Santino Caserta, Maria Eugenia Alvaro, Giuseppa Penna, Manlio Fazio, Fabio Stagno, Alessandro Allegra

**Affiliations:** Division of Hematology, Department of Human Pathology in Adulthood and Childhood “Gaetano Barresi”, University of Messina, via Consolare Valeria, 98125 Messina, Italy; maria_eugenia.alvaro@polime.it (M.E.A.); giusypenna1@gmail.com (G.P.); manliofazio@hotmail.it (M.F.); stagnof@unime.it (F.S.); aallegra@unime.it (A.A.)

**Keywords:** non-Hodgkin lymphoma, chemotherapy, immunotherapy, response rates, gut microbiota, gut microbiota dysbiosis, probiotics, prebiotics, diet, fecal transplantation

## Abstract

Non-Hodgkin B-cell lymphomas are a heterogeneous group of lymphoid malignancies with variable biological behavior, clinical presentation and treatment response. While chemoimmunotherapy remains the cornerstone of their management, growing evidence implicates the gut microbiota as a critical modulator of both lymphomagenesis and therapeutic efficacy. Gut microbiota dysbiosis, characterized by reduced microbial diversity and pathogenic taxonomic shifts, has been observed also in newly diagnosed patients and not just after therapy. This microbial imbalance contributes to mucosal barrier disruption, systemic inflammation, and altered immune responses, affecting treatment outcomes and toxicity profiles. Antibiotic exposure, especially broad-spectrum agents, exacerbates dysbiosis and has been associated with inferior responses to immunochemotherapy and CAR T-cell therapy. Conversely, certain commensal taxa, like Faecalibacterium prausnitzii and Lactobacillus johnsonii, may exert protective effects by preserving mucosal homeostasis and promoting antitumor immunity. Targeted interventions, including prudent antibiotic stewardship, prebiotics, probiotics, dietary modulation, and fecal microbiota transplantation, are under investigation to restore eubiosis and improve clinical outcomes. Preliminary clinical trials suggest a strong correlation between baseline microbiome composition and therapeutic response. Further mechanistic studies and randomized trials are warranted to define the causal role of the microbiome in non-Hodgkin B-cell lymphomas pathophysiology and to develop personalized microbiome-modulating strategies as adjuncts to standard treatment.

## 1. General Background About Non-Hodgkin Lymphomas

Non-Hodgkin lymphomas (NHLs) represent a heterogeneous group of lymphoid malignancies. The global incidence of NHL has increased over the past few decades, with significant geographic variation influenced by environmental, genetic, and infectious factors. According to data from the Global Cancer Observatory (GLOBOCAN) database (2020), NHL is among the ten most common cancers worldwide, with an estimated 544,000 new cases and approximately 260,000 deaths annually. The median age at diagnosis is around 67 years, although specific subtypes may present at younger ages [1,2,3].

NHLs encompass over 60 distinct entities as classified by the World Health Organization (WHO), based on morphological, immunophenotypic, genetic, and clinical criteria. They are broadly categorized into B-cell and T/NK-cell neoplasms, with B-cell lymphomas accounting for approximately 85–90% of all cases [4,5]. Among B-cell NHLs, the most prevalent subtype is diffuse large B-cell lymphoma (DLBCL), constituting about 30–40% of cases [6,7,8,9].

For aggressive B-cell NHLs, particularly DLBCL, the standard first-line therapy is immunochemotherapy with the R-CHOP regimen, which combines rituximab (an anti-CD20 monoclonal antibody) with cyclophosphamide, doxorubicin, vincristine, and prednisone. This regimen induces complete response (CR) rates of approximately 60–75% and overall response rates (ORRs) exceeding 80%. Long-term disease-free survival is achievable in a significant proportion of patients, particularly those with limited-stage disease or favorable prognostic features. Intensified regimens such as dose-adjusted EPOCH-R (etoposide, prednisone, vincristine, cyclophosphamide, doxorubicin, and rituximab) may be used in high-risk cases or specific subtypes such as primary mediastinal B-cell lymphoma, often with improved outcomes [10,11,12,13].

Indolent lymphomas, such as follicular lymphoma (FL), typically follow a relapsing–remitting course. First-line treatment is generally initiated based on symptoms or disease burden, and regimens include rituximab alone for low tumor burden cases or combination therapies such as R-CVP (rituximab, cyclophosphamide, vincristine, and prednisone) or R-CHOP. Bendamustine combined with rituximab (BR) has emerged as a preferred regimen due to comparable efficacy and improved tolerability. In FL, CR rates range from 30 to 50%, with ORR often exceeding 90%; however, long-term remissions are uncommon, and most patients eventually relapse [7]. Mantle cell lymphoma (MCL), due to its aggressive nature, is typically treated with intensive immunochemotherapy, often including high-dose cytarabine followed by autologous stem cell transplantation (ASCT) in eligible patients. BR may also be used in older or less fit individuals. Response rates are generally high (ORR 70–90%), but relapses are frequent [14].

For T-cell lymphomas, CHOP-based regimens remain standard, although responses are often suboptimal, with CR rates below 50% and poor long-term survival. Novel agents and clinical trials are increasingly important in these subtypes [15]. Overall, the first-line treatment of NHL aims to maximize response and prolong survival while minimizing toxicity. Advances in immunotherapy and precision medicine continue to refine therapeutic strategies and improve patient outcomes.

The aim of this narrative review is to explore the topic of gut microbiota dysbiosis in patients with NHL, finding possible dietary interventions and trying to understand if this alteration can have a role on the response to treatments.

In order to reach this target, we searched PubMed, Scopus, and Web of Science databases for articles published in English between January 2015 and June 2025. The following combinations of Medical Subject Headings (MeSH) and keywords were used: “non-Hodgkin lymphoma”; “chemotherapy”; “immunotherapy”; “response rates”; “gut microbiota”; “gut microbiota dysbiosis”; “probiotics”; “prebiotics”; “diet”; “fecal transplantation”. Additional references were identified through manual screening of bibliographies from relevant articles and reviews.

## 2. Adverse Events Related to Treatments: What About Gastrointestinal Toxicity?

Chemotherapy and immunotherapy represent cornerstone treatments in the management of NHL, leading to significant improvements in survival outcomes [16]. However, these modalities are frequently associated with a broad spectrum of adverse effects, both hematological toxicities such as neutropenia, anemia, and thrombocytopenia and non-hematological ones such as gastrointestinal (GI) toxicity [17,18].

GI toxicity encompasses a range of symptoms including nausea, vomiting, mucositis, diarrhea, abdominal pain, and anorexia. These effects may result from direct cytotoxic damage to the rapidly proliferating epithelial lining of the gastrointestinal tract. Regimens such as CHOP or R-CHOP (with rituximab) are commonly used in NHL and have well-documented GI side effects. Diarrhea may also be exacerbated by antibiotic exposure or infectious causes during immunosuppression. Moreover, agents like vincristine can cause autonomic neuropathy leading to ileus or severe constipation [19,20].

Immunotherapy, particularly monoclonal antibodies like rituximab and checkpoint inhibitors used in refractory or relapsed NHL, may compound toxicity by inducing immune-mediated adverse events. Although rituximab is generally well tolerated, it can sometimes induce colitis or hepatitis due to immune dysregulation. Additionally, novel agents such as Chimeric Antigen Receptor (CAR) T-cell therapies and bispecific antibodies introduce unique toxicity profiles, including cytokine release syndrome and immune effector cell-associated neurotoxicity, but also contribute to GI symptoms like diarrhea, nausea, and mucosal inflammation [21].

The interplay between chemotherapy, immunotherapy, and the gut microbiota is gaining attention as a potential modifier of treatment-related GI toxicity. Disruption of microbial diversity—known as intestinal dysbiosis—has been increasingly associated with mucosal injury, inflammation, and exacerbation of gastrointestinal symptoms in patients receiving chemoimmunotherapy. Emerging evidence suggests that maintaining microbiome homeostasis could mitigate toxicity and improve treatment tolerability [22].

In clinical practice, managing GI toxicity involves a combination of prophylactic and supportive strategies, including antiemetics, antidiarrheals, nutritional support, and in some cases, dose adjustments or treatment delays. Close monitoring and early intervention are essential to prevent complications such as dehydration, electrolyte imbalance, or malnutrition. Given the impact of GI and other toxicities on patient outcomes, ongoing research is focused on predictive biomarkers, microbiome modulation, and toxicity-reduction strategies to optimize NHL treatment safety and efficacy [23].

## 3. What Is Gut Microbiota?

The human gut microbiota refers to the diverse community of microorganisms, encompassing bacteria, viruses, fungi, archaea, and protists that reside in various areas of the human body, including the gastrointestinal tract, oral cavity, skin and respiratory tract [24,25].

This complex ecosystem, predominantly localized in the colon, comprises an estimated 10^13^–10^14^ microbial cells and includes over 1,000 different bacterial species, with the majority belonging to the *Firmicutes* and *Bacteroidetes phyla* [12]. Bacteria constitute the most extensively studied component, contributing approximately 99% of the gut microbiota. These microbes are engaged in a symbiotic relationship with the host, influencing numerous physiological functions including nutrient metabolism, vitamin synthesis, protection against pathogens, and maintenance of the intestinal epithelial barrier [13].

The gut microbiota exhibits a highly individualized composition that evolves over the course of a person’s life and is influenced by intrinsic factors like genetic background and immune system activity [14]. External variables—including dietary habits, use of antibiotics and other medications, infections, circadian rhythms, and environmental exposures—also significantly influence the structure and function of the intestinal microbiota [18].

The gut microbiota is essential for maintaining intestinal and systemic homeostasis. By fermenting indigestible carbohydrates, gut bacteria produce short-chain fatty acids (SCFAs) such as butyrate, acetate, and propionate. These SCFAs not only nourish colonic epithelial cells but also exert systemic effects, modulating host metabolism and immune responses. Furthermore, the gut flora supports the maturation and integrity of the gut-associated lymphoid tissue (GALT), which is crucial for immune surveillance and tolerance [19].

In terms of immunomodulation, gut microbiota plays a critical role in both innate and adaptive immune responses. It induces the development of regulatory T cells (Tregs), promotes IgA production by B cells, and influences the secretion of anti-inflammatory cytokines like IL-10. Certain microbial strains, such as *Bacteroides fragilis* and *Clostridium* spp., are particularly effective at promoting an anti-inflammatory milieu, while others, like segmented filamentous bacteria (SFB), drive pro-inflammatory Th17 responses. Dysregulation of this balance—known as dysbiosis—can compromise the intestinal barrier, facilitate the translocation of bacterial components like lipopolysaccharides (LPSs) into systemic circulation, and provoke chronic inflammation.

Dysbiosis has been increasingly recognized as a contributing factor in the pathogenesis of several malignancies, including NHLs. Evidence indicates that microbial imbalance can disrupt immune homeostasis and activate oncogenic pathways. Specifically, microbial products can engage pattern recognition receptors (PRRs), such as Toll-like receptors (TLRs) and NOD-like receptors (NLRs), leading to activation of the NF-κB signaling pathway. This cascade promotes the expression of pro-inflammatory cytokines like TNF-α, IL-1, and IL-6, which contribute to the pro-tumorigenic microenvironment through persistent inflammation, inhibition of apoptosis, and promotion of cellular proliferation [23].

The cumulative effect of microbiota-induced chronic inflammation may alter the equilibrium between cell proliferation and apoptosis, fostering genomic instability and contributing to lymphomagenesis. These immune perturbations provide a fertile ground for the emergence of lymphoid neoplasms, especially in genetically predisposed individuals.

Comparative gut microbiota analysis, utilizing high-throughput sequencing (e.g., 16S rRNA and metagenomic profiling), enables the discrimination between healthy subjects and those with NHL. Patients with B-cell NHL exhibit significant reductions in alpha diversity and a marked shift in microbial composition—specifically, increased *Bacteroidetes* and decreased *Firmicutes*, particularly in aggressive subtypes such as diffuse large B-cell lymphoma [26]. Taxa such as *Barnesiellaceae*, *Coriobacteriaceae*, *Faecalibacterium*, *Christensenella*, and *Sutterella* have been inversely correlated with bloodstream infections in NHL patients. Functional metagenomic evidence further reveals that depletion of *Eubacterium rectale*—a butyrate producer—is a distinguishing hallmark in lymphoma patients, with potential mechanistic roles in attenuating intestinal inflammation and B-cell NF-κB activation [27].

**Figure 1:**
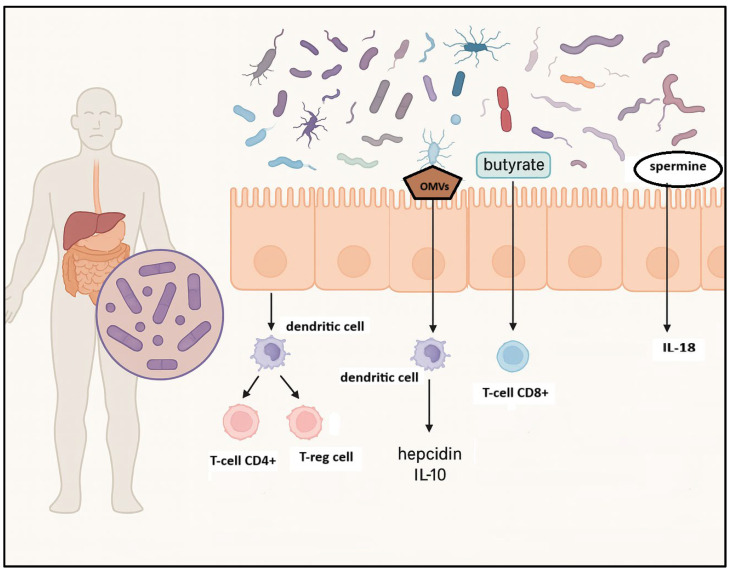
Influence of gut microbiota on innate and adaptive immune response. Gut microbiota influences innate immune responses by regulating the secretion of antimicrobial peptides and modulating natural killer (NK) cell activity; microbial metabolites, such as spermine, lead to IL-18 production; outer membrane vesicles (OMVs) induce IL-10 and hepcidin production. Gut microbiota also shapes adaptive immune responses by encouraging the development of regulatory T cells (T-regs), which promote immune tolerance and activate CD8+ cytotoxic T cells that are critical for B-cell maturation. Created in BioRender. Santino Caserta. (2025) https://app.biorender.com/illustrations/68b5c21594f73e7a5f30080d.

Evidence from preclinical studies supports this association. A landmark investigation by Yamamoto and colleagues utilized a murine model of ataxia–telangiectasia (AT)—a condition marked by defective DNA repair due to mutations in the ATM gene—to examine microbiota-mediated modulation of lymphoma risk [28]. Mice colonized with a limited microbial repertoire exhibited accelerated lymphoma onset, increased oxidative stress, and systemic genotoxicity. Conversely, administration of *Lactobacillus johnsonii* reduced inflammatory and genotoxic markers, suggesting a protective effect against lymphoma development in susceptible hosts [28] (Figure 1).

In vivo investigations in animal models have increasingly elucidated the contributory role of gut microbiota dysbiosis in lymphomagenesis. A notable veterinary study demonstrated that intestinal dysbiosis in dogs and cats—characterized by an increased prevalence of Gram-negative facultative anaerobes such as *Parabacteroides*—is associated with chronic mucosal inflammation and the onset of gastrointestinal lymphoma. In a pilot study of dogs with stage IV multicentric lymphoma, distinct alterations in the fecal microbiota were reported, indicating that lymphoid malignancy correlates with microbiota imbalance [29]. Complementary evidence from chemotherapy studies further supports that interventions aimed at restoring eubiosis can attenuate gastrointestinal damage and systemic immune dysregulation. Although not lymphoma-specific, numerous preclinical models of chemotherapy-induced mucositis—an inflammation-driven gut injury—have shown beneficial effects of probiotics, synbiotics, and prebiotics in mitigating dysbiosis, preserving epithelial integrity, and reducing inflammatory responses [30].

## 4. Gut Microbiota Dysbiosis Due to Chemoimmunotherapy

Chemoimmunotherapy remains the standard of care for B-cell NHL, significantly improving survival outcomes through the synergistic effects of cytotoxic agents and monoclonal antibodies [31]. Despite its clinical efficacy, this therapeutic approach is accompanied by substantial off-target toxicities, particularly within the GI tract [32,33]. A growing body of preclinical and clinical evidence highlights that GI toxicity is not merely a collateral effect but a key driver of intestinal dysbiosis in this patient population [34,35,36]. The integrity of the gut mucosa, a critical interface between the host immune system and the microbiota, is profoundly affected by chemotherapeutic regimens [37,38,39].

The commonly used agents in B-cell NHL—contribute to cumulative damage of the intestinal epithelial lining, inducing direct cytotoxicity to rapidly proliferating epithelial cells, compromising tight junction integrity, and increasing intestinal permeability. Chemotherapy-induced epithelial injury disrupts barrier homeostasis, shifts nutrient availability, and promotes translocation of microbial products—conditions that favor dysbiosis and amplify mucosal inflammation.

Multiple studies have documented a decline in beneficial microbial populations—including *Bifidobacterium* spp., *Lactobacillus*, and *Faecalibacterium prausnitzii*—during and after chemoimmunotherapy. These taxa are known to exert anti-inflammatory effects, support mucosal healing, and maintain regulatory immune responses. Their depletion is paralleled by the overrepresentation of pro-inflammatory species such as *Enterococcus*, *Escherichia coli*, and *Clostridioides difficile*, which further exacerbate GI toxicity. Additionally, rituximab-mediated B-cell depletion reduces secretory IgA levels and impairs antigen-specific immune responses in the gut-associated lymphoid tissue, weakening the mucosal defense against microbial invasion and perpetuating dysbiotic changes [40].

Importantly, intestinal dysbiosis has implications beyond local GI injury, in fact bile acid derivatives, SCFAs and tryptophan metabolites produced by gut microbiota influence systemic immune tone and modulate the tumor microenvironment. Dysregulation of these microbial products may alter immune checkpoint expression, T-cell polarization, and cytokine release, thereby affecting the efficacy of anticancer therapies. Reduced microbial diversity during treatment has been associated with increased GI complications, heightened susceptibility to systemic infections, and inferior clinical outcomes in B-NHL patients [41] (Table 1).

This table summarizes key clinical studies that have evaluated gut microbiome changes in patients with B-cell non-Hodgkin lymphomas undergoing chemotherapy or chemoimmunotherapy. Reported alterations include loss of commensal taxa (*Bifidobacterium*, *Lactobacillus*, *Faecalibacterium prausnitzii*) and expansion of potentially pathogenic species (*Enterococcus*, *Escherichia coli*, *Clostridioides difficile*). These shifts have been associated with gastrointestinal mucosal injury, systemic infections, increased treatment-related toxicities, and inferior therapeutic responses. Abbreviations: R-CHOP, rituximab, cyclophosphamide, doxorubicin, vincristine, prednisone; SCFAs, short-chain fatty acids; GvHD, graft-versus-host disease.

Chemotherapy induces mucosal injury, alters bile acid flux, and reduces luminal carbohydrates, while broad-spectrum and anti-anaerobic antibiotics (e.g., carbapenems, piperacillin–tazobactam, some cephalosporins) deplete SCFA, producing commensals such as Faecalibacterium, Blautia, and other Clostridiales. The resulting loss of microbial diversity and SCFA signaling compromises epithelial barrier integrity, tight junction expression, and colonocyte bioenergetics, amplifying endotoxin translocation and systemic inflammation. In this context, dysbiosis has been associated with higher rates of febrile neutropenia, Clostridioides difficile infection, and bloodstream infections, as well as with attenuated responses to chemoimmunotherapy and cellular therapies in selected cohorts.

The timing and spectrum of antibiotic exposure appear critical. Peri-induction regimens that unnecessarily suppress obligateanaerobes accelerate community collapse (“domination”) by pathobionts such as Enterococcus or Proteobacteria, which correlates with infectious complications and, in transplant candidates, with more severe graft-versus-host disease (GvHD). Conversely, gut-sparing strategies—early de-escalation when cultures are negative, avoidance of redundant double coverage, preference of agents with narrower anaerobic activity when clinically appropriate, and strict duration controls—are consistently linked to preserved diversity. Prophylaxis also matters: while fluoroquinolones or trimethoprim–sulfamethoxazole may reduce specific infections, they can expand the intestinal resistome and select for taxa that sustain dysbiosis; decisions should therefore be individualized to local epidemiology and patient risk.

These antimicrobial effects interact with diet and supportive care. Enteral nutrition favors SCFA recovery and mucosal healing more than parenteral nutrition, and resistant-starch/fermentable-fiber intake can support butyrate-producing consortia when mucosal integrity allows. Probiotics and postbiotics remain promising but require cautious, protocolized use in immunocompromised hosts; the risk–benefit profile varies by strain, preparation, and neutropenic status. In heavily pretreated or transplant settings, microbiome-restorative approaches (e.g., fecal microbiota transplantation under stringent screening) are under investigation for steroid-refractory gastrointestinal GvHD and for decolonization of multidrug-resistant organisms, but they should be confined to experienced centers [42].

Understanding the bidirectional relationship between chemoimmunotherapy-induced GI toxicity and intestinal dysbiosis is crucial for the development of supportive care strategies. Emerging interventions—including dietary modulation, selective prebiotic and probiotic formulations, and fecal microbiota transplantation (FMT)—are under investigation for their potential to preserve mucosal integrity and restore microbial equilibrium. Future research should focus on delineating the molecular mechanisms underlying host-microbiota interactions during therapy, identifying predictive biomarkers of microbiome-related toxicity, and personalizing microbiome-supportive interventions to improve treatment tolerability and efficacy in B-NHL (Table 2).

## 5. Targeted Interventions for Dysbiosis and Influence on Chemoimmunotherapy Response

Disruption of the gut microbial equilibrium, known as dysbiosis, has emerged as a critical factor in both the pathogenesis and treatment responsiveness of NHLs.

Patients newly diagnosed with DLBCL often exhibit distinct alterations in their gut microbiota compared to healthy individuals, characterized by a reduction in alpha diversity and a significantly divergent microbial composition [43]. Notably, this dysbiotic state frequently involves an overrepresentation of Enterobacteriaceae, including species such as *Escherichia coli* and *Citrobacter freundii*, and Enterococcaceae such as *Enterococcus faecium*, whereas healthy individuals demonstrate a higher abundance of beneficial taxa such as Lachnospiraceae, Prevotellaceae, and Coriobacteriaceae. Functional metagenomic analyses suggest that the predominance of Enterobacteriaceae may contribute to opportunistic pathogenesis, including enhanced biofilm formation and mechanisms of antibiotic resistance [44]. Mendelian randomization studies have provided further support for a causal relationship between specific microbial taxa and the risk of DLBCL, implicating genera such as *Ruminococcaceae UCG-002* and *Coprobacter* as potential risk factors, while *Alistipes* and *Turicibacter* may exert protective effects. Similar patterns of microbial disruption, including reduced diversity and specific taxonomic shifts, have been documented in FL, particularly in its gastrointestinal manifestations [45,46,47].

The gut microbiota plays a pivotal role in modulating therapeutic responses across various oncologic treatments, including conventional chemotherapy and advanced therapies such as CAR T-cell therapy, R-CHOP and allogenic hematopoietic stem cell transplantation (HSCT). In patients with DLBCL undergoing R-CHOP chemotherapy, an increased abundance of Enterobacteriaceae correlates with a higher incidence of febrile neutropenia, a complication that is independently associated with reduced progression-free survival, and inferior treatment outcomes; in detail, the 1-year PFS is 70% in patients with high Enterobacteriaceae versus 95% in those with low levels [48]. This microbial pattern may potentiate systemic inflammation, as reflected by elevated circulating levels of interleukin-6 (IL-6) and interferon-gamma (IFN-γ). Conversely, the presence of certain commensal bacteria, such as *Lactobacillus fermentum*, has been associated with a more favorable clinical response, in fact it can enhance host immunity by promoting anti-inflammatory cytokine production (e.g., IL-10) and supporting regulatory T cell activity; these effects may help mitigate systemic inflammation and enhance the tumor-directed immune response. Importantly, chemotherapy itself is a known driver of dysbiosis, leading to marked reductions in microbial richness and diversity [49,50].

In the context of CAR T-cell therapy, the gut microbiota exerts a dual influence on both therapeutic efficacy and toxicity profiles. Research indicates a strong correlation between gut microbial composition and CAR-T cell therapy outcomes; in fact, a study [51] found that specific species of *Clostridia* were associated with achieving a complete response by day 100 following CAR-T treatment.

Accumulating evidence highlights three key domains: antibiotic associations, mechanistic rationale, and exploratory signals with oral vancomycin.

In patients with non-Hodgkin B-cell lymphoma, those who received high-risk antibiotics such as meropenem, cefepime, ceftazidime, and piperacillin–tazobactam during therapy exhibited poorer treatment responses. This adverse outcome was linked to higher abundances of opportunistic pathogens like *Prevotella*, *Veillonella*, and *Enterococcus species*. Conversely, patients who did not receive these high-risk antibiotics demonstrated better treatment responses, which correlated with higher abundances of beneficial bacteria, including *Roseburia*, *Bifidobacterium*, *Lactobacillus*, and *Eubacterium species* [52,53].

CAR-T cell therapy commonly induces significant alterations in the gut microbiota. One study revealed a notable decrease in overall bacterial diversity after CAR-T therapy, with *Firmicutes* becoming more abundant and *Bacteroidetes* decreasing. Additionally, there was an increase in *Enterococcus*, *Lactobacillus*, and *Actinomyces*, while *Bifidobacterium* and *Lachnospira* levels declined. Regarding toxicity, lower levels of *Bifidobacterium* have been significantly correlated with the severity of cytokine release syndrome (CRS), a common toxic effect of CAR-T therapy affecting approximately 80% of patients [40].

Interestingly, oral vancomycin—an antibiotic with minimal systemic absorption—has been shown in preclinical studies to enhance CAR T-cell efficacy and is correlated with increased CAR T-cell expansion in the 100% of patients demonstrated significantly higher CAR T-cell expansion relative to unexposed patients. This phenomenon may be partially explained by the microbiota-modulating effects of vancomycin, which promote tumor-associated antigen (TAA) cross-presentation and endogenous CD8+ T-cell activation. Moreover, FMT experiments involving human-to-mouse transfer have demonstrated that vancomycin-induced shifts in the microbiota, including decreased alpha diversity and enrichment of species such as Enterobacteriaceae and *Akkermansia muciniphila,* can potentiate both CAR and non-CAR T-cell-mediated antitumor immunity [51].

Given these findings, targeted modulation of the gut microbiota represents a promising therapeutic avenue to improve outcomes in NHL.

Prudent antibiotic stewardship is essential, with emphasis on minimizing unnecessary use of broad-spectrum antibiotics during critical phases of treatment to preserve microbial integrity and avoid complications such as febrile neutropenia or diminished CAR T-cell efficacy. Personalized approaches to antimicrobial use, informed by microbial profiling, are likely to enhance therapeutic precision [54].

Additionally, probiotic and prebiotic interventions are under investigation for their potential to enrich beneficial taxa. Certain commensals—such as *Faecalibacterium*, *Prevotella*, *Paraprevotella*, and *Bifidobacterium*—may counterbalance the pathogenic effects of Enterobacteriaceae, while *Lactobacillus johnsonii* has demonstrated the capacity to reduce systemic inflammation and lymphoma incidence in genetically susceptible mouse models. Notably, the strength of evidence varies across these taxa: while *Bifidobacterium* and *Faecalibacterium* have shown emerging supportive data in B-cell NHL and other hematological malignancies, where their depletion has been associated with higher infection risk and inferior clinical outcomes, the evidence for *Prevotella* and *Paraprevotella* remains preliminary and is mainly derived from studies in solid tumors or broader oncology cohorts, thus requiring cautious interpretation in the context of NHL [55,56].

Dietary interventions, including the adoption of Mediterranean-style diets rich in plant-based fibers, have been shown to promote a more favorable microbial profile. While direct evidence of their impact on NHL outcomes remains limited, dietary modulation may represent a supportive measure for maintaining gut eubiosis during treatment [57,58].

Among the most promising strategies is FMT, a therapeutic approach involving the transfer of fecal microbiota from healthy donors to restore microbial balance in recipients. FMT is well-established in the treatment of recurrent *Clostridioides difficile* infection and is being increasingly explored in oncology. In NHL, FMT has shown efficacy in resolving therapy-resistant colitis, particularly cases induced by immune checkpoint inhibitors, which often mimic inflammatory bowel disease and are associated with microbiota dysregulation. In preclinical models and select clinical settings, FMT has also been associated with improved CAR T-cell therapy outcomes. The “donor effect”—the observation that specific donor microbiota engrafts more successfully and yield better clinical responses—highlights the necessity of rigorous donor screening and characterization [34].

Looking ahead, while current data provide compelling associations between microbiota composition and NHL pathophysiology, further large-scale clinical trials are essential to establish causality and define standardized therapeutic protocols. Research should expand beyond bacterial communities to include the mycobiome and virome, which may also play significant roles in immune modulation and tumor biology. These efforts will be instrumental in translating microbiome science into safe, personalized, and mechanistically informed therapies for NHL patients.

Dietary and probiotic interventions aimed at ameliorating chemotherapy-induced dysbiosis in lymphoma patients face multiple limitations. First, chemotherapy-associated mucositis, nausea, anorexia, and gustatory alterations frequently hinder patients’ adherence to prescribed dietary regimens or supplement intake. Second, the profound immunosuppression and compromised mucosal barrier integrity in these patients elevate the risk of bacterial translocation, rendering the safety of probiotic administration—especially strains like *Lactobacillus* and *Bifidobacterium*—a concern in this vulnerable population. Although meta-analyses in cancer patients more broadly have demonstrated that probiotics can reduce the incidence and severity of oral mucositis and diarrhea, these studies often involve heterogeneous cancer types and do not focus specifically on lymphoma patients, limiting generalizability [34]. Moreover, current clinical guidelines (e.g., MASCC/ISOO) for gastrointestinal mucositis prevention emphasize that evidence for dietary and probiotic interventions remains inadequate or conflicting, and no new recommendations have been established due to methodological limitations and low-quality data. Finally, interindividual variability in baseline microbiota composition, tumor biology, and chemotherapy regimens further complicates the prediction of therapeutic response, underscoring the need for personalized intervention strategies that are yet to be validated in rigorous lymphoma-specific trials.

HSCT represents a curative strategy for several hematological malignancies, including selected cases of B-cell non-Hodgkin lymphomas, but its success is frequently limited by infectious complications and graft-versus-host disease (GvHD). Increasing evidence indicates that the intestinal microbiome plays a pivotal role in modulating these outcomes. Conditioning regimens, prophylactic or therapeutic antibiotics, and nutritional alterations profoundly disrupt microbial diversity, leading to a state of dysbiosis that predisposes to systemic inflammation and immune dysregulation [49]. Clinical studies have shown that a marked reduction in microbial richness at the time of engraftment correlates with increased transplant-related mortality and higher incidence of both acute and chronic GvHD. In particular, the loss of commensal taxa such as *Blautia*, *Faecalibacterium*, and other Clostridiales members is consistently associated with a more aggressive GvHD course, whereas intestinal domination by pathogenic organisms, notably *Enterococcus* and *Enterobacteriaceae*, predicts adverse survival outcomes

The microbiota protects the epithelial barrier by producing SCFAs, particularly butyrate and propionate, which drive colonocyte metabolism and promote regulatory T cell development. Reduced SCFA availability after allo-HSCT contributes to epithelial damage, impaired mucosal healing, and a pro-inflammatory milieu favoring GvHD onset. Experimental data further highlight the role of microbial-derived metabolites in modulating donor T-cell alloreactivity, with signaling pathways involving G-protein-coupled receptors and epigenetic regulation of immune effector functions [59].

**Figure 2:**
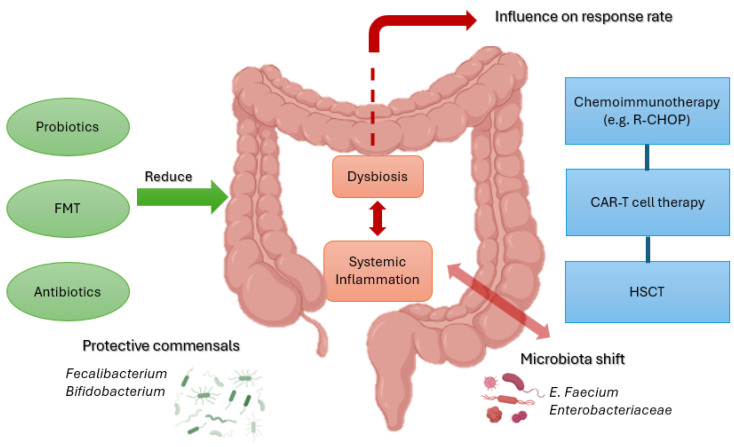
**Schematic representation of the interplay between gut microbiota and treatment responses in B-cell NHL**. Protective taxa (*Faecalibacterium*, *Bifidobacterium*) promote barrier integrity and immune regulation, while dysbiosis with *Enterococcus* and *Enterobacteriaceae* drives inflammation and toxicity. Interventions such as probiotics, FMT, and antibiotic stewardship may restore balance and improve outcomes with chemoimmunotherapy, CAR-T therapy, and HSCT. Created with BioRender. Santino Caserta. (2025) https://app.biorender.com/illustrations/68b5c44de87b2863610e44c6.

From a therapeutic perspective, several microbiome-directed interventions are under investigation. Antibiotic stewardship, aimed at limiting unnecessary exposure to broad-spectrum anaerobic agents, is associated with preserved microbial diversity and reduced GvHD incidence. Moreover, fecal microbiota transplantation (FMT) has emerged as a promising strategy, with preliminary reports documenting restoration of microbial complexity and clinical improvement in patients with steroid-refractory intestinal GvHD [60] (Figure 2).

Overall, the integration of microbiota monitoring and supportive interventions into transplant practice holds the potential to improve outcomes in HSCT recipients. For patients with B-cell lymphomas undergoing allogeneic transplantation, tailoring peri-transplant strategies to preserve or restore microbial equilibrium could mitigate the risk of GvHD while maintaining graft-versus-lymphoma effects. These insights reinforce the importance of considering the gut microbiota as a critical, modifiable factor influencing both treatment-related toxicity and long-term survival in the transplant setting.

## 6. Clinical Trials

Clinical trials exploring the relationship between the gut microbiome and hematologic malignancies have gained momentum in recent years, highlighting the potential influence of microbial composition on treatment response, immune recovery, and therapy-related toxicity. Among these studies, the clinical trial NCT06161896 represents a prospective, observational, single-center cohort, recruiting, study in Europe, enrolling approximately 200 patients newly diagnosed with DLBCL. Its primary purpose is to characterize the baseline gut microbiota and to investigate possible correlations with clinical outcomes, treatment response, and prognosis. Participants provide stool and blood samples, undergo bioelectrical impedance analysis for the assessment of body composition, and complete detailed dietary and lifestyle questionnaires. The study focuses on microbiome diversity and taxonomic composition, correlating these data with treatment efficacy, toxicity, and survival, while also evaluating the impact of potential confounders such as antibiotic exposure, comorbidities, and diet. Recruitment is ongoing, with study completion expected in July 2026 (NCT06161896).

In contrast, the PRIMAL trial (NCT05135351) is a pilot, randomized, double-blind, placebo-controlled, recruiting, interventional study conducted on a small cohort of 30 adult patients with multiple myeloma or lymphoma undergoing ASCT. Its main objective is to assess the feasibility and impact of prebiotic supplementation with resistant starch compared to a maltodextrin placebo, starting ten days before transplantation and continuing until neutrophil engraftment. Unlike NCT06161896, which is purely observational, PRIMAL actively tests a microbiome-modulating intervention and evaluates its effect on gut microbiome diversity, the abundance of beneficial taxa, and serum markers of gut permeability. Secondary outcomes include hospitalization duration, incidence of neutropenic fever, antibiotic exposure, and gastrointestinal symptoms, with exploratory analyses focused on the correlation between dietary patterns and microbial shifts, particularly in taxa such as *Faecalibacterium prausnitzii*, *Ruminococcus*, and *Akkermansia muciniphila*. The trial is actively recruiting at the University of Nebraska Medical Center and includes a dedicated arm for CAR T-cell therapy recipients.

Together, these two studies exemplify complementary strategies for investigating the role of the gut microbiota in lymphoid malignancies. NCT06161896 focuses on describing microbiome–disease associations in a large, newly diagnosed DLBCL cohort without intervention, whereas PRIMAL tests a specific prebiotic approach in a smaller, post-transplant population, emphasizing feasibility and the generation of early efficacy signals. The differences in study design, objectives, and patient populations reflect the diverse approaches currently being used to elucidate the potential therapeutic relevance of the gut microbiome in lymphoma therapy (Table 3).

## 7. Conclusions

The growing body of evidence underscores the gut microbiota as a key player in the pathogenesis, progression, and treatment responsiveness of NHL. Dysbiosis—characterized by reduced microbial diversity and shifts toward pathogenic taxa—has been consistently observed in newly diagnosed NHL patients, particularly those with DLBCL and gastrointestinal follicular lymphoma [1,2,3]. This altered microbial landscape contributes not only to gastrointestinal toxicity and systemic inflammation but also to diminished therapeutic efficacy, notably in the context of chemoimmunotherapy and CAR T-cell therapy [8,12,13].

Chemoimmunotherapeutic agents exacerbate dysbiosis by disrupting epithelial integrity, depleting beneficial commensals, and promoting expansion of opportunistic pathogens such as Enterobacteriaceae and Clostridioides difficile. These changes have been correlated with increased inflammatory cytokines (e.g., IL-6, IFN-γ), febrile neutropenia, and impaired treatment response. Importantly, rituximab-mediated B-cell depletion further impairs mucosal immunity through a reduction in secretory IgA. Similarly, in CAR T-cell therapy, prior exposure to broad-spectrum antibiotics is associated with reduced CAR T-cell expansion, increased toxicity, and inferior overall survival, highlighting the detrimental impact of microbiota disruption on cellular immunotherapy [20,21].

In contrast, a balanced gut microbiome enriched with taxa such as Faecalibacterium prausnitzii, Lactobacillus johnsonii, Paraprevotella, and Akkermansia muciniphila appears to enhance antitumor immunity, support mucosal recovery, and improve therapeutic outcomes. Preclinical and early-phase clinical data suggest that selective antibiotic use (e.g., oral vancomycin) and microbiota-modulating strategies can potentiate CAR T-cell responses by enhancing antigen presentation and endogenous CD8+ T-cell activation. Moreover, the gut microbiota is emerging as a potential prognostic biomarker of treatment response in NHL, with specific microbial signatures correlating with therapeutic success, risk of relapse, and incidence of severe toxicities [52,53].

These insights have spurred the development of microbiota-targeted interventions as adjuncts in NHL management. Strategies under investigation include antibiotic stewardship protocols to minimize unnecessary broad-spectrum exposure, microbial profiling to personalize antimicrobial use, dietary interventions promoting fiber-rich, microbiota-supportive diets, and supplementation with prebiotics and probiotics. FMT is also emerging as a promising therapeutic modality, particularly for managing immune-mediated toxicities and potentially enhancing cellular immunotherapy outcomes: studies emphasize the “donor effect,” underscoring the importance of careful donor selection and microbial characterization to achieve consistent clinical benefit [51].

Clinical trials (NCT0616189 and NCT05135351) are currently exploring correlations between baseline microbiome composition and treatment outcomes, as well as the feasibility of prebiotic supplementation during hematopoietic stem cell transplantation.

In this evolving landscape, artificial intelligence (AI) and machine learning are expected to play a transformative role by enabling integration of high-dimensional microbiome datasets with clinical and molecular variables, improving the ability to predict treatment response, stratify patients by risk, and identify actionable microbial targets. AI-driven models could facilitate the development of microbiota-based prognostic tools and guide real-time decision-making in precision oncology [61].

Moving forward, large-scale prospective trials and mechanistic studies are essential to validate these preliminary findings and establish causality. Furthermore, expanding research beyond bacterial taxa to include the virome and mycobiome may uncover additional dimensions of host–microbiota–tumor interaction [62,63]. Ultimately, integrating microbiome-derived prognostic markers with AI-powered predictive models may optimize therapeutic efficacy, reduce toxicity, and transform the management of non-Hodgkin B-cell lymphomas into a holistic, data-driven, precision-based approach.

## Figures and Tables

**Table 1:** Clinical studies reporting microbiome alterations associated with chemoimmunotherapy in B-cell non-Hodgkin lymphomas.

Therapy Regimen	Patient Population	Reported Microbiome Changes	Clinical Implications
Cyclophosphamide-based chemotherapy	Hematologic malignancies, incl. B-NHL	↓ *Bifidobacterium*, *Lactobacillus*, ↓ diversity	Increased infection risk; poorer survival
Multi-agent chemotherapy	NHL and other hematologic cancers	↓ *Faecalibacterium prausnitzii*; ↑ *Enterococcus*, *E. coli*	Correlation with mucosal injury and bacteremia
Chemotherapy ± antibiotics	Lymphoma and HSCT patients	Loss of commensals; ↑ *Clostridioides difficile*	Higher GI toxicity; GvHD exacerbation
Rituximab-containing chemoimmunotherapy	B-cell NHL	Depletion of IgA-producing *Bifidobacterium* spp.	Impaired mucosal immunity; dysbiosis persistence
R-CHOP regimens	Diffuse large B-cell lymphoma	↓ diversity; depletion of butyrate-producing bacteria	Reduced therapy response; higher relapse risk

**Table 2:** Chemoimmunotherapy related mechanisms leading to gut microbiota dysbiosis in non-Hodgkin B-cell lymphomas: microbial metabolites involved and consequences on immune response.

Step	Mechanism/Effect	Examples
**Therapy effects on gut barrier**	Disruption of mucosal barrier homeostasis and translocation of microbial components → local inflammation	Chemotherapy, radiotherapy, rituximab (B-cell depletion → ↓ secretory IgA)
**Impact on gut microbiota**	Depletion of beneficial microbes and expansion of pro-inflammatory species	↓ Faecalibacterium prausnitzii (anti-inflammatory) ↑ Enterococcus faecalis (pro-inflammatory)
**Consequences of dysbiosis**	Impaired immune responses, increased GI complications and heightened susceptibility to systemic infections	Loss of colonization resistance, overgrowth of Clostridioides difficile
**Microbial metabolites and immune tone**	Dysbiosis alters production of short-chain fatty acids, bile acid derivatives and tryptophan metabolites	SCFAs support Treg differentiation; secondary bile acids regulate inflammation; indoles strengthen epithelial barrier
**Outcome**	Altered systemic immune tone, changes in tumor microenvironment, therapy response or toxicity	Reduced immunotherapy efficacy and increased GI toxicity

**Table 3:** Key aspects of NCT06161896 and NCT05135351 clinical trials about the evaluation of gut microbiota influence on the response to treatments in non-Hodgkin B-cell lymphomas (https://clinicaltrials.gov/, accessed on 27 July 2025).

Trial Identifier	NCT06161896	NCT05135351 (PRIMAL Trial)
**Study Design**	Prospective, observational, single-center cohort study	Pilot, randomized, double-blind, placebo-controlled trial
**Population**	~200 newly diagnosed Diffuse Large B Cell Lymphoma (DLBCL) patients	30 adult patients undergoing autologous stem cell transplantation (ASCT) for multiple myeloma or lymphoma
**Study Aim**	Characterize baseline gut microbiota in DLBCL patients and assess associations with clinical outcomes	Assess feasibility and impact of prebiotic supplementation (resistant starch vs. placebo) on gut microbiota in ASCT patients
**Sample Collection**	Stool and blood samples; body composition via bioelectrical impedance; lifestyle and dietary questionnaires	Stool and blood samples around engraftment; ASA24 dietary survey; gut permeability biomarkers
**Primary Outcomes**	Gut microbiota composition, diversity, and abundance; association with treatment response, toxicity, prognosis	Feasibility of intervention, through the measurement of the percentage of subjects who adhere to >70% of scheduled doses
